# Cross Talk between Lipid Metabolism and Inflammatory Markers in Patients with Diabetic Retinopathy

**DOI:** 10.1155/2015/191382

**Published:** 2015-07-29

**Authors:** Roxanne Crosby-Nwaobi, Irini Chatziralli, Theodoros Sergentanis, Tracy Dew, Angus Forbes, Sobha Sivaprasad

**Affiliations:** ^1^NIHR Moorfields Biomedical Research Centre, London EC1V 2PD, UK; ^2^King's College Hospital NHS Foundation Trust, London SE5 9RS, UK; ^3^Department of Epidemiology and Biostatistics, University of Athens, 11528 Athens, Greece; ^4^King's College London, London SE5 9RS, UK

## Abstract

*Purpose*. The purpose of this study was to examine the relationship between metabolic and inflammatory markers in patients with diabetic retinopathy (DR). *Methods*. 208 adult patients with type 2 diabetes participated in this study and were categorized into (1) mild nonproliferative diabetic retinopathy (NPDR) without clinically significant macular edema (CSME), (2) NPDR with CSME, (3) proliferative diabetic retinopathy (PDR) without CSME, and (4) PDR with CSME. Variable serum metabolic markers were assessed using immunoassays. Multinomial logistic regression analysis was performed. *Results*. Diabetes duration and hypertension are the most significant risk factors for DR. Serum Apo-B and Apo-B/Apo-A ratio were the most significant metabolic risk factors for PDR and CSME. For every 0.1 g/L increase in Apo-B concentration, the risk of PDR and CSME increased by about 1.20 times. We also found that 10 pg/mL increase in serum TNF-*α* was associated with approximately 2-fold risk of PDR/CSME while an increase by 100 pg/mL in serum VEGF concentration correlated with CSME. *Conclusions*. In conclusion, it seems that there is a link between metabolic and inflammatory markers. Apo-B/Apo-A ratio should be evaluated as a reliable risk factor for PDR and CSME, while the role of increased systemic TNF-*α* and VEGF should be explored in CSME.

## 1. Introduction

Diabetic retinopathy (DR) is the most common microvascular complication of diabetes and remains one of the leading causes of adult blindness globally [1]. The prevalence of DR increases with duration of diabetes, and more than 60% of those with type 2 diabetes have some form of DR after 20 years [1]. Early stages of DR (nonproliferative DR or NPDR) are characterized by microaneurysms, dot and blot haemorrhages, and exudates, while the later stages are characterized by retinal neovascularisation and its complications (proliferative DR or PDR) [2]. Diabetic macular edema (DME) may occur at any stage of DR and is characterised by increased vascular permeability and resultant leakage of proteins and lipid exudation (hard exudates) in the central retina (macula) [3]. The two most important visual complications of DR are considered to be DME and PDR [2, 3].

The traditional modifiable risk factors for development and progression of DR and DME are hyperglycaemia and hypertension [4, 5], although it is worthy to note that a recently published Cochrane systematic review reported that there is lack of evidence to support that control of hypertension leads to prevention of DR progression [6]. On the other hand, beneficial effect of intervention to reduce blood pressure with respect to preventing DR was observed in patients who have diabetes for up to 4-5 years [6]. In fact, the known key risk factors only explain 44.6% and 19.5% of total variances in DR and DME, respectively [7]. Therefore, many investigators have explored other modifiable risk factors. An area of renewed interest is the role of dyslipidaemia as a potential risk factor for DR. Several epidemiological studies over the last few decades have evaluated the role of hyperlipidemia in DR by estimating traditional lipid markers, such as serum total cholesterol, triglycerides, low density lipoproteins (LDL), and high density lipoproteins (HDL) with conflicting results. In particular, most studies to date have shown no association between these serum lipid markers and DR but some promising evidence exists, linking these parameters with hard exudates and DME [8].

Interestingly, two recent landmark studies (effect of fenofibrate on the need of laser treatment for diabetic retinopathy (FIELD) and action to control cardiovascular risk in diabetes (ACCORD-Eye)) have shown that fenofibrate could be beneficial in reducing the progression of DR and development of DME [9, 10], as well as the need for laser treatment for sight threatening complications of DR [9]. However, in both studies the effects of these oral medications, like fenofibrate, on DR were unrelated to their effects on blood lipids but may relate to effects on novel pathways, linking dyslipidaemia and DR. Additionally, the traditional lipid profile markers may not be sufficiently sensitive biomarkers for assessing the association between dyslipidaemia and DR [9, 10]. Apart from the lipidic mechanism, recent studies shed light into the nonlipidic mechanism by which fenofibrate exhibits its beneficial action in DR and DME, including antiapoptotic activity, antioxidant and anti-inflammatory activity, neuroprotection, protective effect on blood-retinal-barrier, and potential antiangiogenic effect of fenofibrate in DR [11, 12].

Several reports suggest that the effects of these lipid-lowering agents on DR may be due to their anti-inflammatory effects. There is substantial evidence supporting the role of low grade subclinical inflammation in the pathogenesis of DR, leading to damage to the retinal vasculature and neovascularization [13]. Vascular endothelial growth factor (VEGF) has been implicated in DME pathogenesis by inducing hyperpermeability and therefore vascular leakage, while in PDR it is thought to have angiogenesis activity [14]. In addition, several pro- and anti-inflammatory markers in the serum and ocular fluids have been related to DR and the breakdown of the blood retinal barrier in DME [3]. It is therefore important to evaluate both systemic inflammatory markers and novel serum lipid markers to better understand the interactions of dyslipidaemia and inflammation in PDR and DME.

In this study we explored the relationship of circulating inflammatory markers and novel serum lipid markers that have recently been reported in DR and DME. These include serum adipocytokines, hyperinsulinemia, and apolipoproteins. Adipocytokines, such as adiponectin, leptin, and tumour necrosis factor-alpha (TNF-*α*), influence both lipid metabolism and inflammatory processes and have been linked to both the development and severity of DR [15–18]. Hyperinsulinaemia has been also associated with triglycerides and may precede an abnormal lipid profile, as it is implicated in atherogenesis and considered to be an independent cardiovascular risk predictor [19]. Similarly, Sasongko et al. showed that serum apolipoproteins (apo-A, apo-B, and apo-B/apo-A ratio) are stronger biomarkers of DR compared to traditional lipids [20]. Therefore, estimating adipocytokines and apolipoproteins and correlating them with circulating inflammatory markers in patients with type 2 diabetes with varying severity of DR and DME may provide a better understanding of both the lipid profile and the implication of inflammatory pathways in DR.

In this study, we prospectively evaluated the association and correlation of serum metabolic markers, including adiponectin, leptin, apo-A, and apo-B, in patients with type 2 diabetes with varying grades of DR in a nested case-control study within the South East London-Diabetic Retinopathy Study (SEL-DRS), which is a cross-sectional study, examining the association of DR and a range of metabolic risk factors in patients with diabetes, receiving retinal screening and eye care and residing in three boroughs of South East London [21]. Additionally, we correlated these metabolic markers with previously reported serum pro- and anti-inflammatory markers in DR. The proinflammatory markers included TNF-*α*, sialic acid, interleukin-1*α* (IL-1*α*), interleukin-1*β* (IL-1*β*), and interleukin-6 (IL-6), while the anti-inflammatory markers included interleukin-1 receptor a (IL-1ra), interleukin-4 (IL-4), interleukin-10 (IL-10), and vitamin D. VEGF has been also examined as a pivotal pathogenic factor for both DME and PDR.

## 2. Materials and Methods

A total of 380 patients were recruited from a population-based eye screening program and grouped by severity of DR as follows: NPDR (*n* = 252) and PDR (*n* = 128). 235 participants provided their blood samples. This study included 208 patients, as 27 patients were excluded, due to previous ocular surgery, history of uveitis, and presence of other concomitant ocular or systemic diseases such as glaucoma, cancer, end-stage renal failure, coronary heart diseases, or liver diseases. Patients taking any medications such as corticosteroids or immunosuppressants and those having received intraocular corticosteroids or anti-VEGF agents, known to affect inflammatory markers, were also excluded. The study was conducted in accordance with the tenets of the Declaration of Helsinki and approved by the local institutional review board. Written informed consent was obtained from all participants.

The severity of DR was graded according to the international DR severity scales on standardized 2-field mydriatic fundus colour photographs. Mild DR eyes were categorised as NPDR and the eyes with treated or active retinal neovascularisation were grouped as PDR [22]. Presence of clinically significant macular edema (CSME) was assessed according to ETDRS criteria [23] and categorized as present or absent (CSME and non-CSME). The overall grading was that of the worse eye. Patients were therefore classified into four groups: (a) NPDR and non-CSME (*n* = 115), (b) PDR and non-CSME (*n* = 34), (c) NPDR and CSME (*n* = 45), and (d) PDR and CSME (*n* = 14). The first group was used as a reference group.

Detailed medical and drug history and sociodemographic data for each patient were collected. Demographic characteristics of the enrolled patients included age, gender, race, and duration of DM. Systolic and diastolic pressure were measured in sitting position, after the patient's resting for at least 15 minutes. Hypertension was defined as a systolic blood pressure ≥140 mmHg, a diastolic blood pressure ≥90 mmHg, or treatment with antihypertensive medications. Height and weight were measured to calculate Body Mass Index (BMI).

### 2.1. Blood Sample

The blood samples were centrifuged at 1000 g to assess concentration of serum markers. Each assay was performed according to the manufacturer's instructions. Leptin and adiponectin were assessed using enzyme-linked immunosorbent assay (ELISA), quantitative sandwich enzyme immunoassay technique. The intra-assay coefficients of variation for leptin and adiponectin were 3.3% and 2.5%, respectively. The interassay coefficients of variation for leptin and adiponectin were 5.4% and 6.8%, respectively. Serum apolipoprotein-A (apo-A) and apolipoprotein-B (apo-B) were assessed using a polyethylene glycol enhanced immunoturbidimetric assay (Siemens Healthcare Diagnostics Ltd., Surrey, UK). Intra-assay coefficients of variation for apo-A and apo-B were 1.0% and 1.4%, respectively. Interassay coefficients of variation for apo-A and apo-B were 2.9% and 2.6%, respectively. VEGF was assessed using ELISA, quantitative sandwich enzyme immunoassay technique. Intra-assay and interassay coefficients of variation were 6.7% and 8.8%, respectively. Sialic acid was assessed using sialic acid Quantichrom assay kit (Bioassay Systems, CA, USA). Cytokine (IL-1*α*, IL-1*β*, IL-1ra, IL-4, IL-6, IL-10, and TNF-*α*) concentrations were assessed using milliplex MAP assay based on the Luminex xMAP technology. Intra-assay coefficients of variation for IL-1*α*, IL-1*β*, IL-1ra, IL-4, IL-6, IL-10, and TNF-*α* were 3.3%, 2.3%, 2.1%, 2.9%, 2.0%. 1.6%, and 2.6%, respectively, while interassay coefficients of variation for IL-1*α*, IL-1*β*, IL-1ra, IL-4, IL-6, IL-10, and TNF-*α* were 12.8%, 6.7%, 10.7%, 14.2%, 18.3%, 16.8%, and 13.0%, respectively. 25-OH vitamin D assessment included chemiluminescence immunoassay analysis. The intra- and interassay coefficients of variation were 7.45% and 13.31%, respectively. For each serum factor, out-of-range results lower than the minimum detectable concentration were set equal to 80% of the minimum detectable concentration [24].

### 2.2. Statistical Analysis

Continuous variables were presented as mean (standard deviation (SD)) and categorical variables were presented as absolute (*n*) and relative frequencies (%). Univariate analysis was performed to compare the levels of serum parameters between the four groups; given the deviation from normality, the Kruskal-Wallis test was implemented. For categorical data, Fisher's exact test was used for the comparisons. Secondarily, Spearman's correlation coefficient was calculated to investigate the intercorrelations between the serum factors.

For the multivariate analysis, multinomial logistic regression was performed, with the PDR and CSME status set as the dependent variable. NPDR/non-CSME group was set as the reference category of the model; the associations of serum parameters with the other three groups (PDR/non-CSME, NPDR/CSME, and PDR/CSME) were reported as relative risks (RRs) and 95% confidence intervals (95% CIs). A core model was initially fitted with independent clinical variables proven significant at the univariate analysis. Subsequently, serum parameters that were significantly associated with the PDR and CSME status at the univariate analysis were alternatively introduced as additions to the core model; serum factors were not entered into the model simultaneously given the potential intercorrelations between them in the context of an overall inflammatory status. Statistical analysis was performed using STATA/SE version 13 (Stata Corp., College Station, TX, USA).

## 3. Results

The demographic and clinical data of our sample are shown in Table 1. Univariate analysis showed that male sex differed significantly between the four groups (*p* = 0.032). Patients with PDR (with or without CSME) presented with longer duration of DM in comparison to the other groups (*p* = 0.001). Age and BMI did not correlate with DR severity (*p* > 0.05). In our sample, 13.9% were Asian, 33.7% Black, 50.0% Caucasian, and 2.4% belonged to other races. Ethnicity did not differ between the various studied groups (*p* = 0.077). Hypertension was significantly more frequent in the PDR/CSME group (*p* < 0.001). Significant between-group variability was noted for serum apo-B, apo-B/apo-A ratio, VEGF, and TNF-*α*. No other lipid or inflammatory markers showed any significant difference between groups.

The intercorrelations between the various serum markers are depicted in Table 2. TNF-*α* levels correlated with apo-A, apo-B, VEGF, IL-6, and IL-10. Apo-B correlated with apo-A, IL-1*α*, and IL-6. Leptin correlated with sialic acid, IL-1*β*, and IL-1ra, whereas apo-A correlated with adiponectin and vitamin D. Notably, the inflammatory cytokines (IL-1*α*, IL-1*β*, IL-1ra, IL-4, IL-6, and IL-10) were mutually and strongly correlated.

Table 3 shows the results of the multivariate multinomial logistic regression analysis. Duration of DM was associated with PDR development, as evidenced upon the associations with PDR/non-CSME (RR = 1.10, 95% CI: 1.04–1.16, per 1-year increment) and with PDR/CSME (RR = 1.09, 95% CI: 1.01–1.18, per 1-year increment); on the other hand, the association with NPDR/CSME was not significant (*p* = 0.166). Presence of hypertension was associated with about 3-, 3.5-, and 7-fold increased risk PDR/non-CSME, NPDR/CSME, and PDR/CSME development, respectively. The univariate associations with male sex dissipated at the multivariate approach.

As far as the serum markers are concerned, 0.1 g/L increase in Apo-B concentration was associated with increased risk of PDR/non-CSME, NPDR/CSME, and PDR/CSME, at a comparable degree of about 1.20 times. Accordingly, a 0.1 increase in apo-B/apo-A ratio was associated with increased risk of PDR/non-CSME and NPDR/CSME at 1.18 and 1.24 times, respectively, while for PDR/CME there was a trend of increased risk at 1.25 times, which did not reach statistical significance (*p* = 0.059). In addition, an increase of 100 pg/mL in serum VEGF concentration correlated with CSME occurrence, as evidenced by the two comparable RRs regarding NPDR/CSME and PDR/CSME (RRs about 1.2). Moreover, a 10 pg/mL increase in serum TNF-*α* concentration was associated with increased risk for all evaluated types, namely, PDR/non-CSME (RR = 1.59), NPDR/CSME (RR = 1.68), and PDR/CSME (RR = 2.07).

## 4. Discussion

The principal message of our study is that duration of DM and coexisting hypertension remain the most significant risk factors for PDR and DME. Despite the fact that several landmark studies have shown that control of hypertension significantly reduced the development and progression of DR and DME [25], a recent review reaches a quite different conclusion, reporting that the control of blood pressure has an impact on the prevention of DR only for patients with diabetes up to 4-5 years [6]. Our study shows that hypertension remains a significant problem in patients with visually disabling complications of DR. This observation is in line with previous studies, reporting that each 10 mmHg increase in systolic pressure is associated with an approximately 10% excess risk of early DR and a 15% excess risk of PDR or DME [26].

Regarding serum metabolic markers, we observed an increase in serum apo-B and high apo-B/apo-A ratio to be associated with increased risk of PDR and CSME, confirming previous studies showing that these markers are observed in diabetes with macrovascular and microvascular complications [15–17, 27]. Indeed, Sasongko et al. found that in patients with DM the apo-A level was inversely associated with the presence and the severity of DR, whereas apo-B and the apo-B/apo-A ratio were positively associated with DR [20]. The potential association with DME could not be properly evaluated in the latter study due to the small number of patients with DME [20].

Mechanisms by which apolipoproteins influence microvascular function may be explained by their actions on larger vessels. Apo-A is the structural protein of HDL and better reflects lipid accumulation in peripheral tissues, having anti-inflammatory, antioxidant, and atheroprotective effects. On the contrary, apo-B is associated with the LDL fraction and is a predictor of cardiovascular risk and a proinflammatory mediator [28]. Hu et al. found no statistically significant difference in apo-B levels between mild NPDR and PDR, although low apo-A/apo-B ratio in serum was associated with more severe DR [29]. In our study, 0.1 g/L increase in apo-B serum concentration and 0.1 increase in apo-B/apo-A ratio are associated with an increased risk of PDR/non-CSME, NPDR/CSME, and PDR/CSME by approximately 1.20 times. We suggest that apo-B/apo-A can be used as key lipid biomarker in future studies evaluating role of dyslipidemia in DR. Nonfasting apo-B/apo-A1 ratio is already known to be superior to any of the traditional serum lipid ratios for myocardial infarction [30]. Furthermore, we also postulate that reducing Apo-B levels may have contributed to the positive effect of fenofibrate on PDR and CSME in the FIELD study and this should be investigated in future clinical trials on fenofibrate in DR [9]. The response of apo-B to statins seems to have significant interindividual variations [31].

We also observed that adipocytokines, leptin, and adiponectin were not significantly associated with PDR or CSME. Studies that have investigated these markers in DR have reported conflicting results probably due to differences in ethnic background, case definition, and proportion of patients with advanced DR and on thiazolidinediones [15–17, 27]. The only adipocytokine that was significantly associated with PDR and CSME was TNF-*α*. We found that 10 pg/mL increase of TNF-*α* concentration was associated with about 2-fold increased risk of PDR/CSME. However, TNF-*α* is not only an adipocytokine. Various other stimuli also cascade circulating TNF-*α*, including hyperglycaemia and advanced glycation end product receptors [32]. Many previous studies support the role of circulating TNF-*α* in PDR patients [33, 34]. This multifunctional cytokine induces apoptosis, differentiation, and cell activation and typically cause low grade inflammation [18, 35]. The role of circulating TNF-*α* in CSME is also well supported by cell culture and animal studies that have demonstrated increased permeability of retinal endothelial cells [36]. Furthermore Huang et al. demonstrated that TNF-*α* is critical in the late breakdown of blood retinal barrier in a knockout strain of mice [37].

Serum VEGF was the only other inflammatory cytokine that was found to be elevated in CSME. Interestingly, serum VEGF concentrations were not elevated in PDR despite the fact that vitreous VEGF is significantly higher in PDR than in NPDR eyes indicating local ocular stimuli are responsible for its fundamental role in angiogenesis in PDR [38]. In our study, an increase by 100 pg/mL in serum VEGF concentration correlated with CSME confirming previous report that serum VEGF concentration correlated positively with the disruption of the external limiting membrane and ellipsoid zone in the outer retina [33, 39–41]. Nevertheless, our study results should be interpreted with caution as serum VEGF is not a reliable estimate of circulating VEGF.

Apart from serum TNF-*α* and VEGF, none of the other serum inflammatory markers showed significant differences between DR groups. Several proinflammatory cytokines have been reported to be increased in aqueous and vitreous of patients with DR [33, 41–44]. However, studies investigating serum inflammatory markers show conflicting reports. In addition, there is poor correlation between serum and ocular cytokines in DR [45, 46]. Our study suggests that circulating TNF-*α* may indeed be the link between dyslipidemia and inflammation in patients with DR. Other than being an adipocytokine, TNF-*α* correlates both with other metabolic markers (apo-A and apo-B) and with inflammatory markers (VEGF, IL-6, and IL-10). Interestingly, TNF-*α* correlates with both pro- and anti-inflammatory cytokines suggesting that a TNF-*α* related imbalance of pro- and anti-inflammatory cytokines may indeed result in a low grade inflammatory milieu in patients with PDR and CSME. However, our study results may be only reflecting the presence of other microvascular or macrovascular complications in this high risk group.

Several limitations of the present study should be addressed. Firstly, the cross-sectional design of this study does not permit us to establish a causal relationship between systemic apo-B or TNF-*α* and PDR as well as CSME. Secondly, our study population consisted of individuals, who regularly attended diabetes clinics and were monitored frequently, so the generalizability of the present study could be limited. It would be also valuable to correlate our results with the presence of microalbuminuria or diabetic nephropathy, but no enough data were available. Moreover, we did not have a control group without diabetes, although this was outside the scope of this study. Thirdly, the concentrations of some cytokines showed large variations and the negative findings of serum markers with either DR of DME could be the result of insufficient power of the study samples to detect weaker associations. Finally, we could not exclude the possibility that confounding factors related to other diabetes related complications may have affected our study results. Therefore, our results should not be misinterpreted and they should be examined in larger, prospective studies, evaluating these variables and potential treatment alternatives to modify them.

## 5. Conclusions

In conclusion, duration of DM and hypertension remain key factors for the progression of DR and DME. As far as the serum markers are concerned, further studies should evaluate apo-B/apo-A ratio as a reliable risk factor for PDR and CSME. In addition, the role of increased systemic TNF-*α* and VEGF should be explored especially in CSME given the variations in treatment response to local anti-VEGF agents.

## Figures and Tables

**Table 1:** Demographic characteristics and inflammatory markers in our sample. Bold cells denote statistically significant associations.

	Nonproliferative DR/non-CSME (*n* = 115)	Proliferative DR/non-CSME (*n* = 34)	Nonproliferative DR/CSME (*n* = 45)	Proliferative DR/CSME (*n* = 14)	*p* value
	Mean ± standard deviation				
Age (years)	67.3 ± 12.9	66.4 ± 9.9	67.2 ± 8.6	66.0 ± 11.2	0.772
Duration of DM (years)	13.5 ± 6.4	18.8 ± 8.8	15.2 ± 8.0	17.6 ± 6.9	**0.001**
BMI	30.9 ± 7.8	30.4 ± 6.7	30.2 ± 5.5	31.1 ± 6.9	0.981

	*N* (%)				
Male sex	61 (53.0)	25 (73.5)	30 (66.7)	5 (35.7)	**0.032**
Hypertension	41 (38.0)	22 (64.7)	31 (68.9)	10 (76.9)	**<0.001**

	Mean ± standard deviation				
Leptin (ng/mL)	27.2 ± 33.9	22.7 ± 24.4	21.8 ± 21.7	27.9 ± 20.6	0.391
Adiponectin (ng/mL)	10389.3 ± 6373.1	10566.7 ± 6165.8	11646.2 ± 7270.7	15712.1 ± 8702.9	0.179
Sialic acid (*μ*M)	3365.8 ± 778.2	3139.7 ± 396.2	3052.3 ± 527.9	3613.7 ± 729	0.051
ApoA (g/L)	1.4 ± 0.5	1.5 ± 0.3	1.6 ± 0.3	1.6 ± 0.5	0.203
ApoB (g/L)	0.5 ± 0.5	0.8 ± 0.2	0.9 ± 0.3	0.8 ± 0.2	**0.0001**
ApoB/ApoA	0.39 ± 0.32	0.54 ± 0.18	0.57 ± 0.22	0.54 ± 0.17	**0.0003**
Vitamin D (ng/mL)	10.5 ± 10	9.5 ± 5.8	11.4 ± 5.9	10.1 ± 5.2	0.135
VEGF (pg/mL)	335.5 ± 235.3	431.0 ± 270.4	451.9 ± 283.6	508.7 ± 349.4	**0.017**
IL-1*α* (pg/mL)	12.2 ± 14.8	12.0 ± 12.7	16.7 ± 34.2	9.3 ± 6.7	0.734
IL-1*β* (pg/mL)	1.0 ± 1.3	0.7 ± 0.2	0.8 ± 0.7	0.9 ± 0.9	0.968
IL-1ra (pg/mL)	13.9 ± 22.6	10.8 ± 10.8	11.7 ± 16.9	11.3 ± 13.2	0.949
IL-4 (pg/mL)	10.0 ± 13.5	6.4 ± 11.4	8.5 ± 11.0	8.2 ± 12.7	0.052
IL-6 (pg/mL)	6.5 ± 14.9	3.6 ± 8	6.0 ± 10.0	3.2 ± 4.5	0.380
IL-10 (pg/mL)	3.6 ± 8.8	4.8 ± 12.3	3.6 ± 6.8	2.7 ± 3.3	0.821
TNF-*α* (pg/mL)	11.5 ± 9.4	15.3 ± 8.3	15.2 ± 11.2	17 ± 13.8	**0.003**

DM: diabetes mellitus; BMI: Body Mass Index; DR: diabetic retinopathy; CSME: clinically significant macular edema; VEGF: vascular endothelial growth factor; IL: interleukin.

**Table 2:** Intercorrelations between the measured serum parameters. Bold cells denote statistically significant associations.

	Leptin (ng/mL)	Adiponectin (ng/mL)	Sialic acid (*μ*M)	ApoA (g/L)	ApoB (g/L)	Vitamin D (ng/mL)	VEGF (pg/mL)	IL-1*α* (pg/mL)	IL-1*β* (pg/mL)	IL-1ra (pg/mL)	IL-4 (pg/mL)	IL-6 (pg/mL)	IL-10 (pg/mL)	TNF-alpha (pg/mL)
Leptin (ng/mL)														
Adiponectin (ng/mL)	+0.095 (*p* = 0.174)													
Sialic acid (*μ*M)	**+0.212** **(p = 0.002)**	−0.054 (*p* = 0.443)												
ApoA (g/L)	+0.036 (*p* = 0.607)	**+0.269** **(p = 0.001)**	−0.062 (*p* = 0.378)											
ApoB (g/L)	+0.026 (*p* = 0.716)	+0.064 (*p* = 0.306)	+0.020 (*p* = 0.774)	**+0.392** **(p < 0.0001)**										
Vitamin D (ng/mL)	−0.123 (*p* = 0.110)	+0.077 (*p* = 0.321)	−0.140 (*p* = 0.071)	**+0.191** **(p = 0.013)**	+0.122 (*p* = 0.114)									
VEGF (pg/mL)	−0.016 (*p* = 0.815)	−0.084 (*p* = 0.230)	+0.043 (*p* = 0.538)	−0.046 (*p* = 0.509)	+0.012 (*p* = 0.868)	−0.047 (*p* = 0.542)								
IL-1*α* (pg/mL)	+0.021 (*p* = 0.773)	+0.103 (*p* = 0.148)	+0.053 (*p* = 0.418)	+0.121 (*p* = 0.088)	**+0.170** **(p = 0.016)**	+0.143 (*p* = 0.069)	+0.005 (*p* = 0.995)							
IL-1*β* (pg/mL)	**+0.170** **(p = 0.016)**	−0.024 (*p* = 0.740)	+0.020 (*p* = 0.774)	−0.041 (*p* = 0.528)	−0.028 (*p* = 0.691)	−0.150 (*p* = 0.056)	−0.018 (*p* = 0.803)	**+0.147** **(p = 0.041)**						
IL-1ra (pg/mL)	**+0.164** **(p = 0.020)**	−0.038 (*p* = 0.592)	+0.120 (*p* = 0.091)	−0.069 (*p* = 0.335)	+0.081 (*p* = 0.254)	+0.002 (*p* = 0.998)	+0.017 (*p* = 0.815)	**+0.435** **(p < 0.0001)**	**+0.486** **(p < 0.001)**					
IL-4 (pg/mL)	+0.077 (*p* = 0.278)	+0.029 (*p* = 0.681)	−0.012 (*p* = 0.868)	−0.001 (*p* = 0.986)	+0.035 (*p* = 0.626)	+0.096 (*p* = 0.226)	+0.001 (*p* = 0.987)	**+0.144** **(p = 0.046)**	**+0.310** **(p < 0.0001)**	**+0.331** **(p < 0.0001)**				
IL-6 (pg/mL)	+0.086 (*p* = 0.222)	+0.004 (*p* = 0.954)	+0.036 (*p* = 0.610)	+0.065 (*p* = 362)	**+0.241** **(p = 0.001)**	+0.039 (*p* = 0.621)	+0.081 (*p* = 0.250)	**+0.280** **(p = 0.0001)**	+0.072 (*p* = 0.319)	**+0.378** **(p < 0.0001)**	**+0.210** **(p = 0.003)**			
IL-10 (pg/mL)	+0.136 (*p* = 0.051)	−0.024 (*p* = 0.731)	+0.055 (*p* = 0.433)	+0.053 (*p* = 0.413)	+0.111 (*p* = 0.112)	−0.016 (*p* = 0.841)	+0.088 (*p* = 0.209)	**+0.207** **(p = 0.003)**	**+0.266** **(p = 0.0001)**	**+0.273** **(p = 0.0001)**	**+0.299** **(p < 0.0001)**	**+0.225** **(p = 0.001)**		
TNF-alpha (pg/mL)	−0.070 (*p* = 0.321)	−0.042 (*p* = 0.552)	+0.007 (*p* = 0.927)	**+0.160** **(p = 0.023)**	**+0.312** **(p < 0.0001)**	+0.120 (*p* = 0.122)	**+0.175** **(p = 0.012)**	+0.136 (*p* = 0.056)	+0.007 (*p* = 0.926)	+0.122 (*p* = 0.086)	+0.128 (*p* = 0.075)	**+0.381** **(p < 0.0001)**	**+0.175** **(p = 0.012)**	

**Table 3:** Results of the multivariate multinomial logistic regression analysis. Bold cells denote statistically significant associations.

Variable	Category/increment	PDR/non-CSME versus ref.^*∗*^		NPDR/CSME versus ref.^*∗*^		PDR/CSME versus ref.^*∗*^	
		RR (95% CI)	*p* value	RR (95% CI)	*p* value	RR (95% CI)	*p* value
Core model: clinical variables							
Male sex	Male versus female	2.08 (0.85–5.09)	0.106	1.54 (0.72–3.28)	0.263	0.30 (0.08–1.11)	0.072
Duration of diabetes	One-year increase	**1.10 (1.04–1.16) **	**0.001**	1.04 (0.99–1.09)	0.166	**1.09 (1.01–1.18) **	**0.037**
Hypertension	Yes versus no	**2.91 (1.25–6.80) **	**0.013**	**3.52 (1.65–7.48) **	**0.001**	**6.83 (1.70–27.39) **	**0.007**
Serum parameters alternatively introduced to the model^†^							
Apo-B	0.1 g/L increase	**1.20 (1.06–1.36)**	**0.003**	**1.27 (1.14–1.42)**	**<0.001**	**1.20 (1.01–1.43)**	**0.039**
Apo-B/Apo-A	0.1 increase	**1.18 (1.01–1.38)**	**0.038**	**1.24 (1.08–1.42)**	**0.002**	1.25 (0.99–1.59)	0.059
VEGF	100 pg/mL increase	1.15 (0.99–1.35)	0.072	**1.17 (1.02–1.35)**	**0.026**	**1.24 (1.02–1.50)**	**0.034**
TNF-alpha	10 pg/mL increase	**1.59 (1.01–2.50)**	**0.046**	**1.68 (1.10–2.56)**	**0.015**	**2.07 (1.20–3.59)**	**0.009**

PDR: proliferative diabetic retinopathy; CSME: clinically significant macular edema; RR: relative ratio; CI: confidence interval; VEGF: vascular endothelial growth factor.

^*∗*^ref.: nonproliferative/non-CSME patients, set as reference category; †: adjusted for the parameters included in the core model (male sex, duration of disease, and hypertension).

## References

[1] Klein R., Klein B. E. K., Moss S. E., Cruickshanks K. J. (1994). The Wisconsin epidemiologic study of diabetic retinopathy. XIV. Ten-year incidence and progression of diabetic retinopathy. *Archives of Ophthalmology*.

[2] Antonetti D. A., Klein R., Gardner T. W. (2012). Diabetic retinopathy. *The New England Journal of Medicine*.

[3] Aiello L. P., DCCT/EDIC Research Group (2014). Diabetic retinopathy and other ocular findings in the diabetes control and complications trial/epidemiology of diabetes interventions and complications study. *Diabetes Care*.

[4] Chatziralli I. P., Sergentanis T. N., Keryttopoulos P., Vatkalis N., Agorastos A., Papazisis L. (2010). Risk factors associated with diabetic retinopathy in patients with diabetes mellitus type 2. *BMC Research Notes*.

[5] Simó R., Hernández C. (2015). Novel approaches for treating diabetic retinopathy based on recent pathogenic evidence. *Progress in Retinal and Eye Research*.

[6] Do D. V., Wang X., Vedula S. S. (2015). Blood pressure control for diabetic retinopathy. *The Cochrane Database of Systematic Reviews*.

[7] Xie J., Fenwick E. K., Taouk Y., et al (2014). Relative importance and contribiton of risk factors for diabetic retinopathy and macular edema. *Journal of Diabetes & Metabolism*.

[8] Chang Y.-C., Wu W.-C. (2013). Dyslipidemia and diabetic retinopathy. *Review of Diabetic Studies*.

[9] Keech A., Simes R. J., Barter P. (2005). Effects of long-term fenofibrate therapy on cardiovascular events in 9795 people with type 2 diabetes mellitus (the FIELD study): randomised controlled trial. *The Lancet*.

[10] ACCORD Study Group, ACCORD Eye Study Group, Chew E. Y. (2010). Effects of medical therapies on retinopathy progression in type 2 diabetes. *The New England Journal of Medicine*.

[11] Wong T. Y., Simó R., Mitchell P. (2012). Fenofibrate—a potential systemic treatment for diabetic retinopathy?. *The American Journal of Ophthalmology*.

[12] Simó R., Roy S., Behar-Cohen F., Keech A., Mitchell P., Wong T. Y. (2013). Fenofibrate: a new treatment for diabetic retinopathy. Molecular mechanisms and future perspectives. *Current Medicinal Chemistry*.

[13] Tomić M., Ljubić S., Kaštelan S. (2013). The role of inflammation and endothelial dysfunction in the pathogenesis of diabetic retinopathy. *Collegium Antropologicum*.

[14] Simó R., Sundstrom J. M., Antonetti D. A. (2014). Ocular anti-VEGF therapy for diabetic retinopathy: the role of VEGF in the pathogenesis of diabetic retinopathy. *Diabetes Care*.

[15] Kato K., Osawa H., Ochi M. (2008). Serum total and high molecular weight adiponectin levels are correlated with the severity of diabetic retinopathy and nephropathy. *Clinical Endocrinology*.

[16] Yilmaz M. I., Sonmez A., Acikel C. (2004). Adiponectin may play a part in the pathogenesis of diabetic retinopathy. *European Journal of Endocrinology*.

[17] Uckaya G., Ozata M., Bayraktar Z., Erten V., Bingol N., Ozdemir I. C. (2000). Is leptin associated with diabetic retinopathy?. *Diabetes Care*.

[18] Joussen A. M., Doehmen S., Le M. L. (2009). TNF-*α* mediated apoptosis plays an important role in the development of early diabetic retinopathy and long-term histopathological alterations. *Molecular Vision*.

[19] Arrants J. (1994). Hyperinsulinemia and cardiovascular risk. *Heart and Lung*.

[20] Sasongko M. B., Wong T. Y., Nguyen T. T. (2011). Serum apolipoprotein AI and B are stronger biomarkers of diabetic retinopathy than traditional lipids. *Diabetes Care*.

[21] Crosby-Nwaobi R. R., Sivaprasad S., Amiel S., Forbes A. (2013). The relationship between diabetic retinopathy and cognitive impairment. *Diabetes Care*.

[22] Wilkinson C. P., Ferris F. L., Klein R. E. (2003). Proposed international clinical diabetic retinopathy and diabetic macular edema disease severity scales. *Ophthalmology*.

[23] Kinyoun J., Barton F., Fisher M., Hubbard L., Aiello L., Ferris F. (1989). Detection of diabetic macular edema. Ophthalmoscopy versus photography—early treatment diabetic retinopathy study report number 5. The ETDRS Research Group. *Ophthalmology*.

[24] Provatopoulou X., Georgiadou D., Sergentanis T. N. (2014). Interleukins as markers of inflammation in malignant and benign thyroid disease. *Inflammation Research*.

[25] Sjølie A. K., Klein R., Porta M. (2008). Effect of candesartan on progression and regression of retinopathy in type 2 diabetes (DIRECT-Protect 2): a randomised placebo-controlled trial. *The Lancet*.

[26] Gallego P. H., Craig M. E., Hing S., Donaghue K. C. (2008). Role of blood pressure in development of early retinopathy in adolescents with type 1 diabetes: prospective cohort study. *British Medical Journal*.

[27] Jung C.-H., Kim B.-Y., Mok J.-O., Kang S.-K., Kim C.-H. (2014). Association between serum adipocytokine levels and microangiopathies in patients with type 2 diabetes mellitus. *Journal of Diabetes Investigation*.

[28] Hill S. A., McQueen M. J. (1997). Reverse cholesterol transport—a review of the process and its clinical implications. *Clinical Biochemistry*.

[29] Hu A., Luo Y., Li T. (2012). Low serum apolipoprotein A1/B ratio is associated with proliferative diabetic retinopathy in type 2 diabetes. *Graefe's Archive for Clinical and Experimental Ophthalmology*.

[30] McQueen M. J., Hawken S., Wang X. (2008). Lipids, lipoproteins, and apolipoproteins as risk markers of myocardial infarction in 52 countries (the INTERHEART study): a case-control study. *The Lancet*.

[31] Boekholdt S. M., Hovingh G. K., Mora S. (2014). Very low levels of atherogenic lipoproteins and the risk for cardiovascular events: a meta-analysis of statin trials. *Journal of the American College of Cardiology*.

[32] Yokoi M., Yamagishi S.-I., Takeuchi M. (2005). Elevations of AGE and vascular endothelial growth factor with decreased total antioxidant status in the vitreous fluid of diabetic patients with retinopathy. *British Journal of Ophthalmology*.

[33] Koleva-Georgieva D. N., Sivkova N. P., Terzieva D. (2011). Serum inflammatory cytokines IL-1beta, IL-6, TNF-alpha and VEGF have influence on the development of diabetic retinopathy. *Folia Medica*.

[34] Paine S. K., Sen A., Choudhuri S. (2012). Association of tumor necrosis factor *α*, interleukin 6, and interleukin 10 promoter polymorphism with proliferative diabetic retinopathy in type 2 diabetic subjects. *Retina*.

[35] Daviaud D., Boucher J., Gesta S. (2006). TNF*α* up-regulates apelin expression in human and mouse adipose tissue. *The FASEB Journal*.

[36] Aveleira C. A., Lin C.-M., Abcouwer S. F., Ambrósio A. F., Antonetti D. A. (2010). TNF-*α* signals through PKC*ζ*/NF-*κ*B to alter the tight junction complex and increase retinal endothelial cell permeability. *Diabetes*.

[37] Huang H., Gandhi J. K., Zhong X. (2011). TNF*α* is required for late BRB breakdown in diabetic retinopathy, and its inhibition prevents leukostasis and protects vessels and neurons from apoptosis. *Investigative Ophthalmology and Visual Science*.

[38] Aiello L. P. (1996). Vascular endothelial growth factor and the eye. Past, present and future. *Archives of ophthalmology*.

[39] Jain A., Saxena S., Khanna V. K., Shukla R. K., Meyer C. H. (2013). Status of serum VEGF and ICAM-1 and its association with external limiting membrane and inner segment-outer segment junction disruption in type 2 diabetes mellitus. *Molecular Vision*.

[40] Baharivand N., Zarghami N., Panahi F., Dokht Ghafari M. Y., Fard A. M., Mohajeri A. (2012). Relationship between vitreous and serum vascular endothelial growth factor levels, control of diabetes and microalbuminuria in proliferative diabetic retinopathy. *Clinical Ophthalmology*.

[41] Schlingemann R. O., Van Noorden C. J. F., Diekman M. J. M. (2013). VEGF levels in plasma in relation to platelet activation, glycemic control, and microvascular complications in type 1 diabetes. *Diabetes Care*.

[42] Celebiler Cavusoglu A., Bilgili S., Alaluf A. (2007). Vascular endothelial growth factor level in the serum of diabetic patients with retinopathy. *Annals of Ophthalmology*.

[43] Cheung C. M. G., Vania M., Ang M., Chee S. P., Li J. (2012). Comparison of aqueous humor cytokine and chemokine levels in diabetic patients with and without retinopathy. *Molecular Vision*.

[44] Funatsu H., Yamashita H., Noma H. (2005). Aqueous humor levels of cytokines are related to vitreous levels and progression of diabetic retinopathy in diabetic patients. *Graefe's Archive for Clinical and Experimental Ophthalmology*.

[45] Hernández C., Segura R. M., Fonollosa A., Carrasco E., Francisco G., Simó R. (2005). Interleukin-8, monocyte chemoattractant protein-1 and IL-10 in the vitreous fluid of patients with proliferative diabetic retinopathy. *Diabetic Medicine*.

[46] Yoshimura T., Sonoda K.-H., Sugahara M. (2009). Comprehensive analysis of inflammatory immune mediators in vitreoretinal diseases. *PLoS ONE*.

